# Bilateral Representation of Sensorimotor Responses in Benign Adult Familial Myoclonus Epilepsy: An MEG Study

**DOI:** 10.3389/fneur.2021.759866

**Published:** 2021-10-26

**Authors:** Teppei Matsubara, Seppo P. Ahlfors, Tatsuya Mima, Koichi Hagiwara, Hiroshi Shigeto, Shozo Tobimatsu, Yoshinobu Goto, Steven Stufflebeam

**Affiliations:** ^1^Department of Radiology, Athinoula A. Martinos Center for Biomedical Imaging, Massachusetts General Hospital, Boston, MA, United States; ^2^Harvard Medical School, Boston, MA, United States; ^3^Research Fellow of Japan Society for the Promotion of Science, Tokyo, Japan; ^4^International University of Health and Welfare, Otawara, Japan; ^5^Graduate School of Core Ethics and Frontier Sciences, Ritsumeikan University, Kyoto, Japan; ^6^Epilepsy and Sleep Center, Fukuoka Sanno Hospital, Fukuoka, Japan; ^7^Division of Medical Technology, Department of Health Sciences, Graduate School of Medical Sciences, Kyushu University, Fukuoka, Japan; ^8^Department of Orthoptics, Faculty of Medicine, Fukuoka International University of Health and Welfare, Fukuoka, Japan; ^9^Department of Physiology, School of Medicine, International University of Health and Welfare, Okawa, Japan

**Keywords:** benign adult familial myoclonus epilepsy (BAFME), sensorimotor cortex, ipsilateral somatosensory-evoked field, C-reflex, transcallosal connectivity

## Abstract

Patients with cortical reflex myoclonus manifest typical neurophysiologic characteristics due to primary sensorimotor cortex (S1/M1) hyperexcitability, namely, contralateral giant somatosensory-evoked potentials/fields and a C-reflex (CR) in the stimulated arm. Some patients show a CR in both arms in response to unilateral stimulation, with about 10-ms delay in the non-stimulated compared with the stimulated arm. This bilateral C-reflex (BCR) may reflect strong involvement of bilateral S1/M1. However, the significance and exact pathophysiology of BCR within 50 ms are yet to be established because it is difficult to identify a true ipsilateral response in the presence of the giant component in the contralateral hemisphere. We hypothesized that in patients with BCR, bilateral S1/M1 activity will be detected using MEG source localization and interhemispheric connectivity will be stronger than in healthy controls (HCs) between S1/M1 cortices. We recruited five patients with cortical reflex myoclonus with BCR and 15 HCs. All patients had benign adult familial myoclonus epilepsy. The median nerve was electrically stimulated unilaterally. Ipsilateral activity was investigated in functional regions of interest that were determined by the N20m response to contralateral stimulation. Functional connectivity was investigated using weighted phase-lag index (wPLI) in the time-frequency window of 30–50 ms and 30–100 Hz. Among seven of the 10 arms of the patients who showed BCR, the average onset-to-onset delay between the stimulated and the non-stimulated arm was 8.4 ms. Ipsilateral S1/M1 activity was prominent in patients. The average time difference between bilateral cortical activities was 9.4 ms. The average wPLI was significantly higher in the patients compared with HCs in specific cortico-cortical connections. These connections included precentral-precentral, postcentral-precentral, inferior parietal (IP)-precentral, and IP-postcentral cortices interhemispherically (contralateral region-ipsilateral region), and precentral-IP and postcentral-IP intrahemispherically (contralateral region-contralateral region). The ipsilateral response in patients with BCR may be a pathologically enhanced motor response homologous to the giant component, which was too weak to be reliably detected in HCs. Bilateral representation of sensorimotor responses is associated with disinhibition of the transcallosal inhibitory pathway within homologous motor cortices, which is mediated by the IP. IP may play a role in suppressing the inappropriate movements seen in cortical myoclonus.

## Introduction

**Figure 1:**
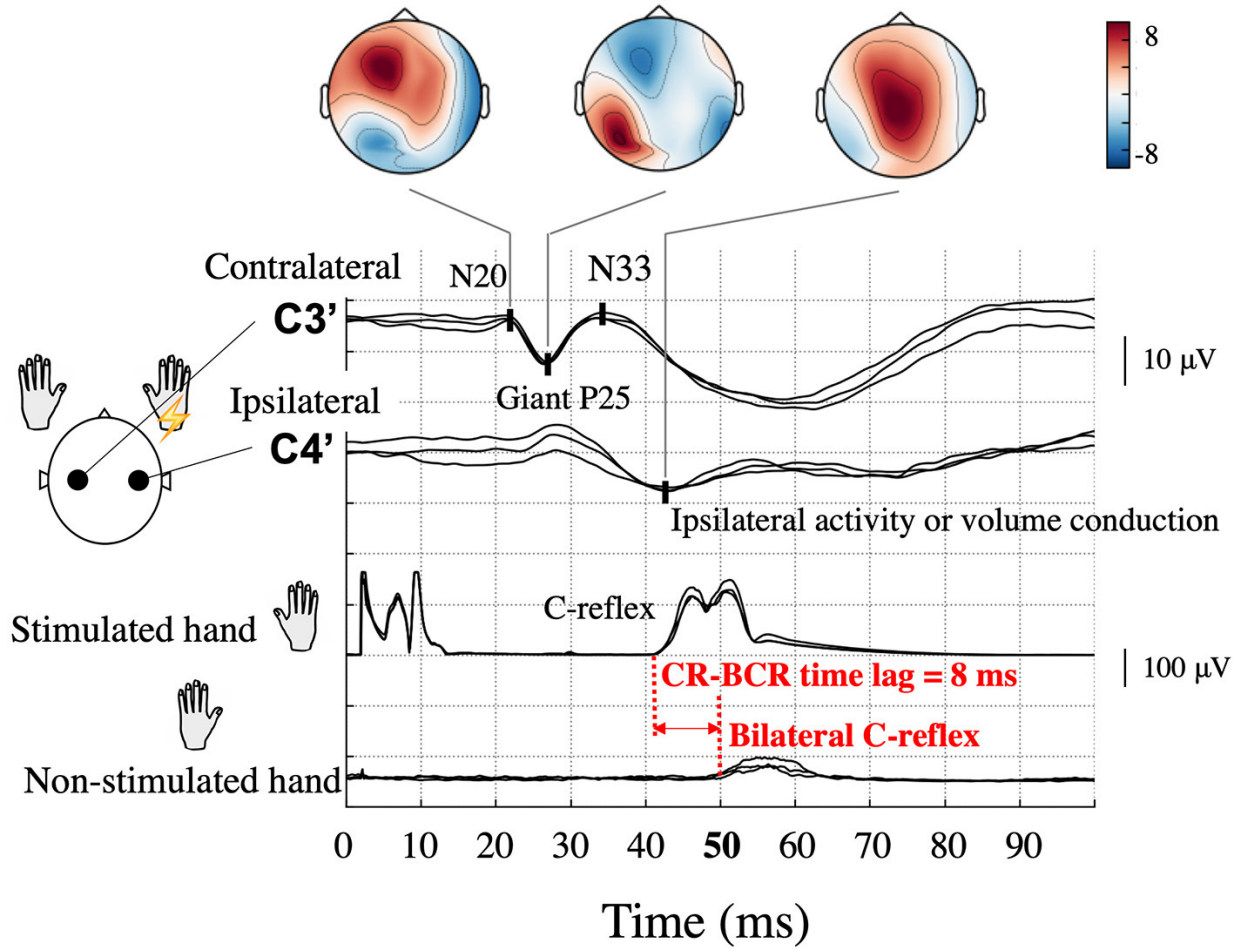
Typical somatosensory-evoked potential and electromyography recordings following right median nerve stimulation in a patient (Patient 2) with bilateral C-reflex (BCR). High amplitude P25-N33 components (giant P25) are prominent at the contralateral hand sensory area (C3′) electrode, and similar potentials can be observed at the corresponding ipsilateral electrode (C4′). Topographical maps are shown according to components. Ipsilateral activity is unreliable because of interference from the contralateral giant component. The onset of the C-reflex (CR), which is shown in the stimulated hand, was 42 ms, whereas that of the BCR in the non-stimulated hand was 50 ms; thus, the CR-BCR time lag was 8 ms.

Conventional neurophysiological studies have demonstrated that one type of myoclonus originates from the cerebral cortex (1–3). This type of myoclonus is often referred to as cortical reflex myoclonus, seen in various diseases such as juvenile myoclonic epilepsy, progressive myoclonic epilepsy, post-anoxic myoclonus, corticobasal degeneration, Alzheimer's disease, advanced Creutzfeldt-Jacob diseases, metabolic encephalopathy and Celiac disease (1–3). Cortical reflex myoclonus manifests two major neurophysiological characteristics that are due to primary sensorimotor cortex (S1/M1) hyperexcitability (4–7), namely, the giant somatosensory-evoked potential/field (SEP/SEF) and the C-reflex (CR; Figure 1). Giant SEP/SEF refers to the enhanced amplitudes of S1/M1 activation. CR, or long-loop reflex, is the EMG response associated with myoclonic jerks that is recorded from the stimulated hand at a latency of around 45 ms after stimulation of the median nerve in the wrist (5, 8, 9). These characteristics are thought to result from a release effect that causes increased excitability at central synapses along the pathway that begins from peripheral input to the spinal cord, the contralateral nucleus of thalamus, contralateral S1/M1, corticospinal tract, anterior horn cell, and finally to the stimulated hand muscle (9).

In some patients, an EMG response is demonstrated in the non-stimulated (opposite) hand muscle (bilateral C-reflex, BCR). In a few reports of small case series, the latency difference between CRs in the stimulated and non-stimulated hand muscle was ~10 ms (CR-BCR time lag, Figure 1) (3, 10–12). This time lag is compatible with the conduction time of the impulse between the homologous S1/M1 via the corpus callosum (13, 14). However, the significance and exact pathophysiology of this BCR is yet to be established. In previous BCR studies, which all employed EEG, identifying ipsilateral cortical activity has been challenging because cross-talk from the giant SEP component in the contralateral hemisphere can overshadow a true response in the ipsilateral hemisphere (4). Source localization methods hold a promise of better dissociating ipsilateral and contralateral activity and thus may help to reveal the precise pathophysiology of BCR. Given the close link between the processes involved in cortical myoclonus and those producing epilepsy (2, 15), the same mechanisms of spread of cortical excitation may also be important in some forms of seizure generalization.

The aim of the present study was to examine the pathophysiological mechanism underlying the early spread of cortical excitation in the bilateral representation of myoclonic jerks in patients showing BCR. The presence of a CR-BCR time lag suggests that (1) ipsilateral cortical activity (i.e., the same side as the stimulated hand) exists, and (2) the time lag between the contralateral (i.e., opposite side to the stimulated hand) and ipsilateral cortical activity corresponds to the CR-BCR time lag. We hypothesized that in patients with BCR (1) bilateral S1/M1 activity can be detected by magnetoencephalography (MEG) source estimation and (2) functional connectivity will be enhanced transcallosally between the contralateral S1/M1 and homologous ipsilateral regions.

## Materials and Methods

### Subjects

Five patients with cortical reflex myoclonus (age 40–70 years, mean age 54.9 years) with BCR were identified from the MEG database of epilepsy patients (January 2005–June 2019) at Kyushu University. All patients had benign adult familial myoclonus epilepsy (BAFME) that fulfilled criteria based on clinical and electrophysiological findings (12, 16, 17) and were treated with antiepileptic drugs (AEDs). The diagnosis was made by board-certified epileptologists (TMa and HS). The cardinal features of BAFME consisted of six items (18, 19): (1) autosomal dominant inheritance; (2) cortical tremor, which consists of continuous, distal, fine twitches of the hands that resemble essential tremor; (3) infrequent generalized seizure; (4) features of cortical reflex myoclonus demonstrated in electrophysiological studies; (5) lack of cognitive decline or other neurological symptoms during the early stage of the clinical course; and (6) lack of clear progression, which impairs activities of daily living in the early stage of the clinical course. Electrophysiological studies included resting-state scalp EEG, SEP, CR, and jerk-locked back averaging (JLA) (20). SEP/CR/BCR was performed as a screening; the recording procedure is described in section SEP and CR/BCR below. JLA, time-locked pre-myoclonus cortical activity (3, 21, 22) showed no preceding positive spikes in any of the patients. Cortical myoclonus in Celiac diseases and corticobasal degeneration shows no preceding positive spikes because of repetitive nature and high frequency of the myoclonus (23), therefore JLA may sometimes show no activity in cortical tremor. Patient demographic data are shown in Table 1. A total of 15 healthy controls (HCs, age 25–51 years, mean 34.6) were recruited. All subjects gave informed consent, according to the approval by the Ethical Committee of Kyushu University Hospital.

**Table 1:** Patient demographic information.

**Subject**	**Age/sex**	**Family history**	**EEG findings**	**Age at seizure onset**	**Frequency of generalized seizures**	**Cortical tremor**	**Medication**
Patient 1	40.2/F	Family A	Generalized Photoparoxysmal discharges	33	<1/y	BUE	LEV
Patient 2	70.2/F	Family A	Generalized Photoparoxysmal discharges	42	<1/y	BUE	PHT, LEV
Patient 3	56.1/F	Family B	W.N.L.	None	None	BUE < BLE	CZP
Patient 4	54.2/F	Sporadic	Generalized Photoparoxysmal discharges	23	6/y	BUE	LEV, CZP
Patient 5	53.7/M	Family B	Generalized Photomyogenic response*	None	None	BUE	CZP

*BUE, bilateral upper extremities; BLE, bilateral lower extremities; LEV, levetiracetam; PHT, phenytoin; CZP, clonazepam.*

*Lower extremity myoclonus accompanied with photic stimulation.

### Stimulus

The median nerve trunk in the wrist was unilaterally stimulated with an electric square pulse of 0.2 ms duration in separate sessions. The stimulus was applied using a pair of electrodes placed on the skin 3 cm apart with the cathode proximal to the anode. Stimulus intensity was just above the motor threshold of the abductor pollicis brevis muscle. Stimulus parameters were different for SEP/CR/BCR and SEF recordings because SEP/CR/BCR was used for diagnostic confirmation of cortical reflex myoclonus (i.e., long latency), whereas SEF was measured as part of routine clinical workup for epilepsy patients, irrespective of seizure type (i.e., short latency) (24).

### MEG Recordings

MEG signals were recorded using a whole-head 306 channel sensor array (Elekta, Neuromag) with 102 identical triple-sensor elements. Before recording, four head-position-indicator coils were attached to the subject's head. Anatomical landmarks (nasion and bilateral preauricular points) and scalp shape using ~200 head-surface points were digitized to construct a three-dimensional head coordinate system co-registered with MRI. At the beginning of the recording session, the subject's head position was measured with respect to the sensor array. The recording was performed in a quiet magnetically-shielded room while subjects lay in a supine position with their head positioned inside the helmet-shaped sensor array. The sampling rate was 1 kHz with an online band-pass filter of 0.1–330 Hz for Patients 1, 2, and 4. For Patients 3 and 5, the sampling rate was 5 kHz and the data were downsampled to 1 kHz. A spatiotemporal signal space separation method was applied to the data offline to reduce external artifact signals (25).

### MRI Scan

High-resolution three-dimensional MRI images were acquired using a 3-T clinical scanner (Philips Healthcare, Best, the Netherlands). The whole brain was scanned using a T1-weighted fast-field echo sequence using the following parameters: repetition time = 8.2 ms; echo time = 3.8 ms; flip angle = 8°; 190 sagittal slices; and 1.0-mm isotropic voxels without a gap. Cortical surface reconstructions were obtained using FreeSurfer (26).

### Data Analysis

#### SEP and CR/BCR

CR/BCR and giant SEP were confirmed as a screening prior to MEG recording on a separate day. Surface EMG was recorded bilaterally from a pair of cup electrodes placed 3 cm apart on the belly of the abductor pollicis brevis muscle of the stimulated side and on the other muscles that produced involuntary jerks (CR and BCR). For EEG recording, multiple cup electrodes were placed on the scalp, which included the hand sensory areas (C3′ and C4′) and Fz according to the International 10–20 system. Electrode impedance was maintained below 5 kOhm. All electrodes were referenced to linked earlobe electrodes. EEG and rectified EMG data were fed into a computer and averaged using the stimulus pulse as the trigger. SEP and CR/BCR were obtained by stimulating the median nerve in the wrist using electric shocks, which were delivered at a rate of 1 Hz in all patients. The passband for EEG was set to 0.5–200 Hz. Components of giant SEPs were identified by corresponding components of normal SEPs (27): an initial negative peak was defined as N20, a following positive peak as P25, and a second negative peak as N33. An SEP was judged as a giant SEP when the amplitude of the component corresponding to N33 measured from the P25 peak was higher than 8.4 μV (3, 27). CR/BCR was identified when the EMG amplitude showed a prominent rise from baseline (3). Data from at least two separate sessions of 200 responses each were obtained to confirm reproducibility. Typical giant SEP and CR/BCR are illustrated in Figure 1. It should be noted that ipsilateral activity in the SEP may mimic the giant component coming from the contralateral hemisphere (4).

Although all patients demonstrated CR in both arms during left and right median nerve stimulation, BCR was observed in the left, right, or both arms. For each arm that showed BCR, we also measured the time lag between CR and BCR for onset-to-onset (CR-BCR time lag, Figure 1).

#### SEF

For Patients 1, 2, and 5, and all HCs, the interstimulus interval (ISI) was constant at 449 ms. For Patient 4 the ISI was 997 ms, and for Patient 3, stimuli were presented using a 2,000-ms ISI with a 250 ms jitter. SEFs were obtained by averaging ~120 responses offline. Trials exceeding 4,000 fT/cm in amplitude on a gradiometer and 4,000 fT on a magnetometer were excluded before averaging. Artifacts, such as eye blinks, other eye movements, and epileptic spikes, were carefully excluded by visual inspection. Raw MEG data were band-pass filtered at 1–120 Hz. The analysis time window was 200 ms, which included a pre-stimulus baseline of 100 ms. Amplitudes were measured from baseline. Because of a lack of clear criteria for giant SEF, activity was judged as a giant SEF when P25m source activity (see section Source estimation), normalized by the N20m amplitude, was greater than the average + 2 standard deviations (SD) of that of HCs. For Patient 5, SEF data from the left median nerve stimulation were not recorded because of a technical reason. During the SEF recording, simultaneous EMG was measured in two of the patients (Patients 2 and 4).

#### Source Estimation

Source current distributions for the SEFs were estimated using the minimum-norm estimate (MNE) (28, 29) and noise-normalized using the dynamic statistical parametrical mapping (dSPM) method (30). The cortical source space consisted of 8,196 dipoles. The forward solution was computed using a Boundary Element Method mesh by tessellating the inner skull surface (31). Source orientation was partially constrained to be perpendicular to the cortex, with the loose orientation constraint parameter set to 0.2 (32). The noise covariance matrix was estimated from the baseline period. Source time courses for each region of interest (ROI) were obtained by averaging the estimated dSPM time for all source dipoles within the ROI. The MNE solutions were regularized by setting the parameter for the expected signal-to-noise ratio to 3.

#### Delineating the Primary Sensorimotor Areas

In BAFME patients, SEF typically includes contralateral N20m and P25m. The N20m represents the normal response from S1 [specifically, it represents the thalamocortical tract (33)]. Indeed, in our BAFME patients, the amplitude and latency of the N20m in the sensor space were not significantly different from those in the HCs (64.5 ± 42.7 fT/cm vs. 56.8 ± 24.0 fT/cm for amplitude, *p* = 0.44; 21.3 ± 1.3 vs. 22.1 ± 1.5 ms for latency, *p* = 0.07). In contrast, the contralateral P25m represents a giant response from S1 and/or M1. Although the generator of the giant P25m is yet to be established, Mima et al. (6) reported equivalent current dipoles (ECDs) of P25m were located at the precentral motor cortex (Brodmann area 4) in 4 patients with cortical reflex myoclonus among 5 patients. A giant P25m may reflect the tangential component of an enlarged radial generator source located at the crown of the precentral gyrus (6, 34). However, P25m in HCs is rarely recognizable, which is likely related to the orientation of the generator source; few reports on P25m (P22m) have been published to date (35, 36).

**Figure 2:**
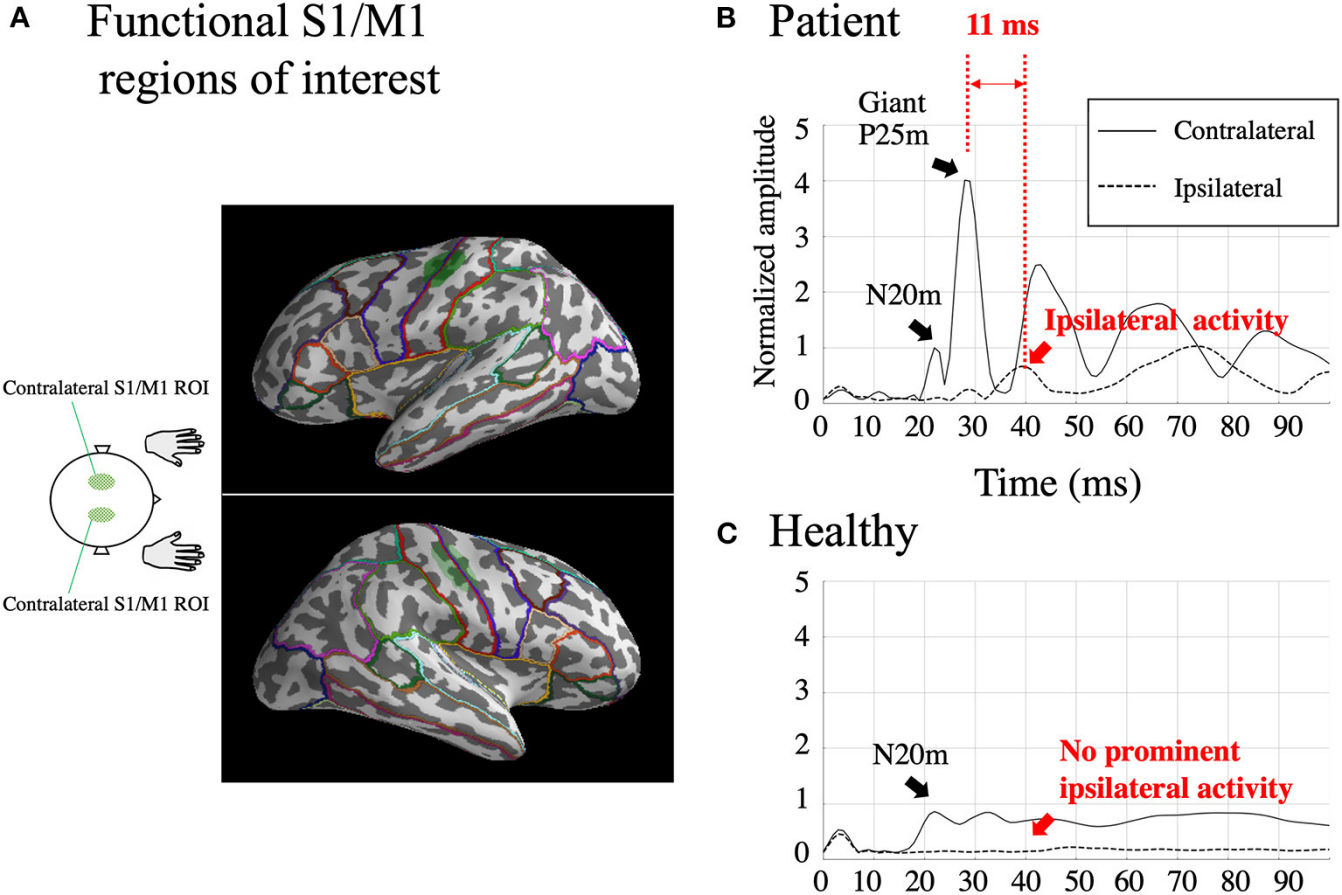
Functional S1/M1 regions of interest (ROIs; green shaded areas) are shown in one subject [Patient 2; **(A)**]. Borders of anatomical regions obtained from the Desikan-Killiany Atlas are shown in different colors. Estimated somatosensory-evoked field source activity within functional S1/M1 regions of interest in response to right median nerve stimulation from one patient who showed BCR [Patient 2; **(B)**] and the grand-average of healthy controls **(C)**. Black lines: contralateral activity; dotted lines: ipsilateral activity. The figure shows a prominent contralateral giant P25m component. The ipsilateral activity, shown within 50 ms, peaks 11 ms later than the contralateral giant component. The amplitudes were individually normalized by contralateral N20m amplitudes.

Because our primary concern is hyperexcitability of S1/M1 in patients with BCR, we first defined functional ROIs to represent S1/M1. The functional S1/M1 ROIs were delineated individually for both the patients and HCs based on the cortical activations at the peak of the contralateral N20m. The S1/M1 ROIs were located at the border of the anatomical central sulcus (Figure 2A, green shaded areas). On average, the delineated S1/M1 ROIs contained 49.8 ± 22.1 dipoles in the patients and 50.2 ± 16.4 dipoles in HCs (*p* = 0.28). Because of the spatial point-spread function, even for a focal source, the MNE solution can extend across sulcal walls (37, 38); therefore, it is reasonable to assume that ROIs determined using the N20m can represent both S1 and M1 activity. The S1/M1 ROIs were obtained for each stimulus to the left and right median nerve in all patients except Patient 5. In Patient 5, who did not have a recording of the left median nerve stimulation, the right S1/M1 ROI was defined using the location homologous to that obtained from the right median nerve stimulation.

The two homologous S1/M1 ROIs were used to investigate ipsilateral activity evoked by the median nerve stimuli. For patients with BCR, ipsilateral activity was identified when the activity increased prominently above baseline and peaked at 20–50 ms. For comparison, the amplitude of any ipsilateral activity at 20–50 ms was investigated in HCs. This time window was set based on the finding (Table 2) that the average onset time of BCR was 46 ± 1.8 ms (range 43–48 ms). Ipsilateral activity in patients with BCR should be observed after the first cortical activity (contralateral N20m) and before BCR (i.e., within the 20–50 ms latency window). In contrast, HCs were expected to show no significant ipsilateral activity within 50 ms because only a few studies have demonstrated physiological ipsilateral activity in SEF within this time range (39–41) due to signal weakness (42). Focusing on the activity within 50 ms also helps to exclude the possibility of top-down input from secondary somatosensory cortex S2, which displays initial activation at around 60–70 ms after stimulation (43).

**Table 2:** SEP/CR/BCR and SEF results.

**Subject**	**Stimulation side**	**SEP/CR/BCR**						**SEF**				
		**Presence of giant SEP**	**P25 latency (ms)**	**Presence of BCR**	**Onset of CR (ms)**	**Onset of BCR (ms)**	**CR-BCR time lag (ms)**	**Presence of giant SEF**	**P25m latency (ms)**	**Normalized amplitude of ipsilateral activity**	**Latency of ipsilateral activity** **(ms)**	**Time difference between P25m and ipsilateral activity (ms)**
Patient 1	Rt	–	24	–	39	N.A.	N.A.	+	27	0.34	37	10
	Lt	–	24	+	37	47	10	+	27	0.67	37	10
Patient 2	Rt	+	27	+	40	48	8	+	28	0.66	39	11
	Lt	–	26	–	39	N.A.	N.A.	+	31	0.67	41	10
Patient 3	Rt	+	23	+	39	46	8	+	26	0.72	33	7
	Lt	+	23	+	39	47	8	+	26	0.48	35	9
Patient 4	Rt	+	23	+	35	44	9	+	26	0.49	38	12
	Lt	+	22	+	35	43	8	+	28	0.36	38	10
Patient 5	Rt	–	26	+	39	47	8	+	27	0.76	34	7
	Lt	+	24	+	39	48	9	N.A.	N.A.	N.A.	N.A.	N.A.
Mean, SD (7 arms)			24.0 ± 1.8		37.6 ± 2.0	46.0 ± 1.8	8.4 ± 0.8		26.9 ± 0.9	0.59 ± 0.15 (*p* <0.0001)	36.3 ± 2.3 (*p* = 0.4)	9.4 ± 1.9

*SEP, somatosensory-evoked potential; SEF, somatosensory-evoked field; CR, C-reflex; BCR, bilateral C-reflex; Rt, right; Lt, left; SD, standard deviation*.

#### Neural Synchrony

We calculated two indices of neural synchrony: the intertrial phase coherence (ITC), which represents phase synchronization with respect to the stimuli, and the weighted phase-lag index (wPLI), which is a measure of inter-areal phase synchrony. To compute these measures, we convoluted the epoched time series with a dictionary of complex Morlet wavelets (each spanning seven cycles) in the frequency range of 13–120 Hz in 1-Hz steps. ITC is a measure of the variance in phase across trials and thus reflects the temporal stability of oscillatory activity (44–47). ITC values range from 0 to 1, where 0 represents no phase-locking and 1 represents perfectly synchronized phase angles across trials.

The wPLI is based on the phase-lag index (PLI) (48), which defines connectivity as the absolute value of the average sign of phase angle differences. PLI detects consistent phase differences between signals. The wPLI was proposed by Vinck et al. (49) to improve specificity as well as robustness to noise and volume conduction-related artifacts. By weighting each phase difference according to the magnitude of the lag, phase differences around zero only marginally contribute to the calculation of the wPLI. This procedure reduces the probability of detecting false positive connectivity in the case of volume conducted noise sources with near-zero phase-lag and increases the sensitivity of detecting phase synchronization (49). Given that patients with BCR manifested the giant component, which spread widely to the ipsilateral hemisphere, wPLI is well-suited to reveal artifact-free connectivity between the contralateral and ipsilateral hemispheres.

Both indices were computed using MNE-python (28, 50). ITCs were evaluated in the homologous S1/M1 ROIs (see section Delineating the primary sensorimotor areas) to determine the optimal time-frequency window within which the wPLI was evaluated. Because ITC provides information that is independent of inter-areal connectivity (i.e., wPLI), its use in determining the time-frequency window of interest avoids selection bias for choosing the time-frequency window for the wPLI analysis.

The wPLI was computed (a) between the contralateral and ipsilateral S1/M1 ROIs, (b) between the contralateral S1/M1 ROI vs. 64 anatomical regions from the Desikan-Killiany (DK) Atlas parcellation (51) (Figure 2A), and (c) between all pairs (all-to-all connectivity) among the 64 anatomical regions. In the all-to-all connectivity, all interhemispheric pairs of regions were included as well as intrahemispheric pairs in the contralateral hemisphere; however, intrahemispheric connectivity within the ipsilateral hemisphere was omitted because ipsilateral activity was expected to be too weak to yield reliable results.

### Statistical Analysis

For between-group comparisons of the amplitude and latency of ipsilateral activity, we applied the Mann-Whitney U test, except for the amplitude and latency of P25m because some HCs lacked an identifiable P25m. The amplitude of dSPM is affected by background brain activity, which is expected to differ between BAFME patients and HCs because the background activity of BAFME patients is significantly slower (17). Therefore, S1/M1 dSPM source waveforms were normalized by the peak amplitudes of the contralateral N20m, which were comparable across the two groups.

The wPLI was averaged over the 30–50 ms and 30–100 Hz time-frequency window, determined from the results of the ITC analysis (see **Figure 4A** in the Results section). This frequency window was assumed to represent the reafferent cortical activity that occurs in a large cortical network to allow integration of external somatosensory stimuli (52). For wPLI values, we used the Mann-Whitney U test and applied correction for multiple comparisons based on the false discovery rate using a threshold of 0.05. All statistics were conducted using MNE-Python and related libraries.

## Results

### SEP and CR/BCR

In the five patients, a giant SEP was observed in response to stimulation of six of the 10 arms (Table 2). The average latency of the P25 was 24.2 ± 1.5 ms (10 arms).

All patients showed CR in both arms, and BCR was further observed in eight arms. The average onset time of CR was 38.0 ± 1.8 ms (10 arms). Of the eight arms that showed BCR, MEG data were available for seven of them. The average onset times of CR and BCR over these seven arms were 37.6 ± 2.0 and 46.0 ± 1.8 ms, respectively, and the average CR-BCR time lag was 8.4 ± 0.8 ms (Table 2). This indicated that the onset latency of the long-loop reflex in the non-stimulated hand was 8 ms longer than that in the stimulated side.

In the two patients (Patients 2 and 4) whose EMG was recorded during SEF recording, individual CR-BCR time lags were similar to those obtained prior to the MEG study. This indicated that the CR-BCR time lag was reproducible over separate days.

### Ipsilateral and Contralateral SEF Activity

**Figure 3:**
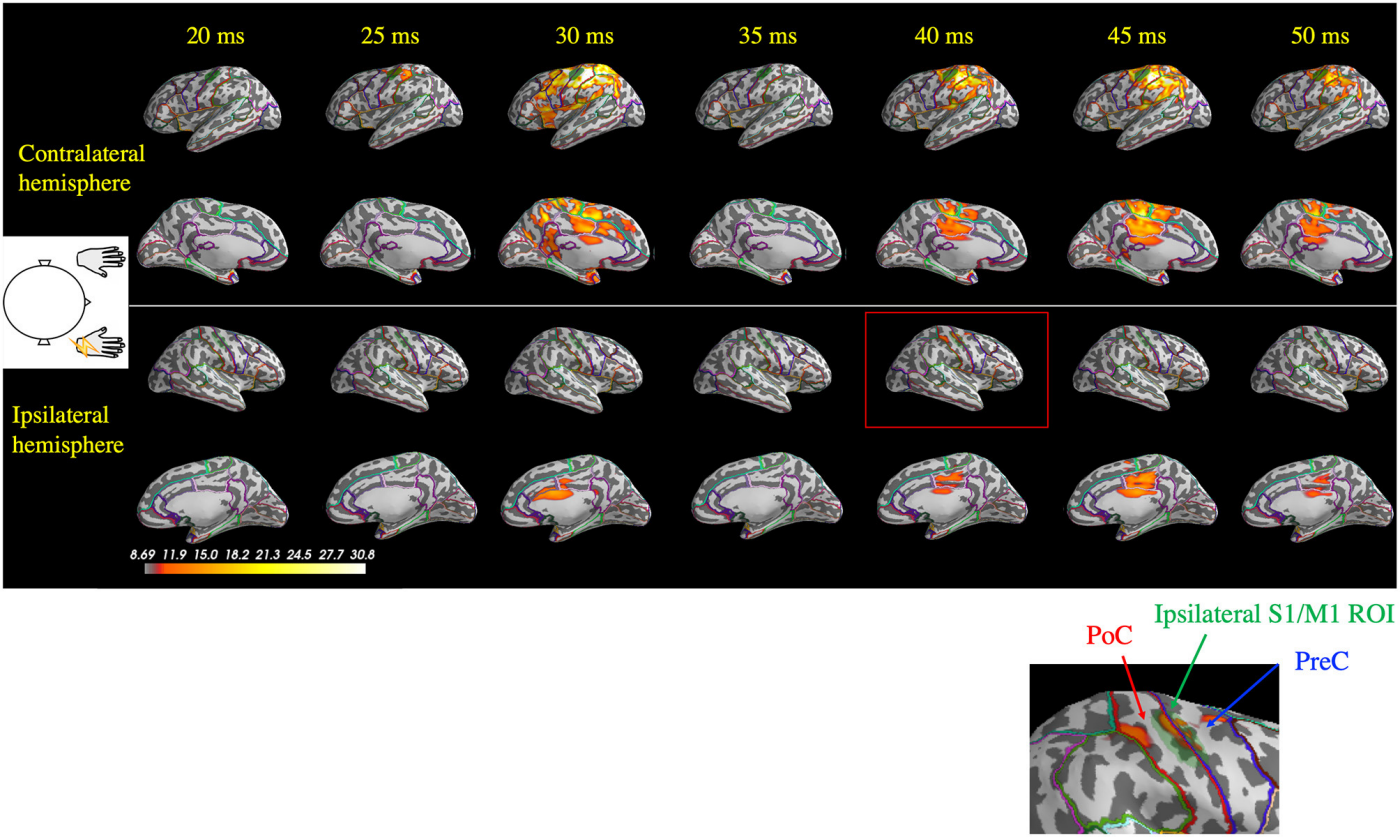
Spatiotemporal distribution of the estimated cortical activity that generated the somatosensory-evoked field following right median nerve stimulation in one patient (Patient 2). Ipsilateral activity (right hemisphere) at around 40 ms (red rectangle) was observed exactly in the ipsilateral S1/M1 region of interest (ROI; green shaded area, highlighted in the inset figure). At 45 ms, contralateral activity remained prominent, whereas the ipsilateral did not. PoC, postcentral gyrus; PreC, precentral gyrus.

For all the seven stimulated arms in the patients that showed BCR, the MEG data revealed a giant component of P25m in the contralateral hemisphere (Table 2). The average latency of the P25m was 26.9 ± 0.9 ms (7 arms). Ipsilateral activity showed a peak latency of 36.3 ± 2.3 ms, which had a smaller amplitude than that of contralateral activity. The amplitude of ipsilateral activity of the patients was significantly larger than that of the HCs (*p* < 0.0001). In the patients, the difference in the time delay between the peak latencies of the contralateral P25m and ipsilateral activity was 9.4 ± 1.9 ms, which was similar to the CR-BCR time lag (see section SEP and CR/BCR). SEF activity of the functional S1/M1 ROIs of a representative patient (Patient 2) and the corresponding grand-average activity of HCs are presented in Figure 2B. In this patient, the contralateral P25m activity was giant, whereas the ipsilateral activity was prominent with an 11-ms delay in its peak from P25m activity (Figure 2B). No prominent ipsilateral activity was observed in HCs (Figure 2C). Typical spatiotemporal distribution of estimated cortical activity (Patient 2, right median nerve stimulation) is presented in Figure 3. Ipsilateral activity was prominent around 40 ms, exactly in the ipsilateral S1/M1 ROI (highlighted in the inset figure as a green shaded area). The time course of the ipsilateral activity (Figure 3, lower two panels) was distinct from that of the contralateral activity (upper two panels), which suggested that the observed ipsilateral activity in the MEG source estimates is likely to be a true response and not due to artifactual cross-talk from the giant contralateral activity.

### Neural Synchrony

**Figure 4:**
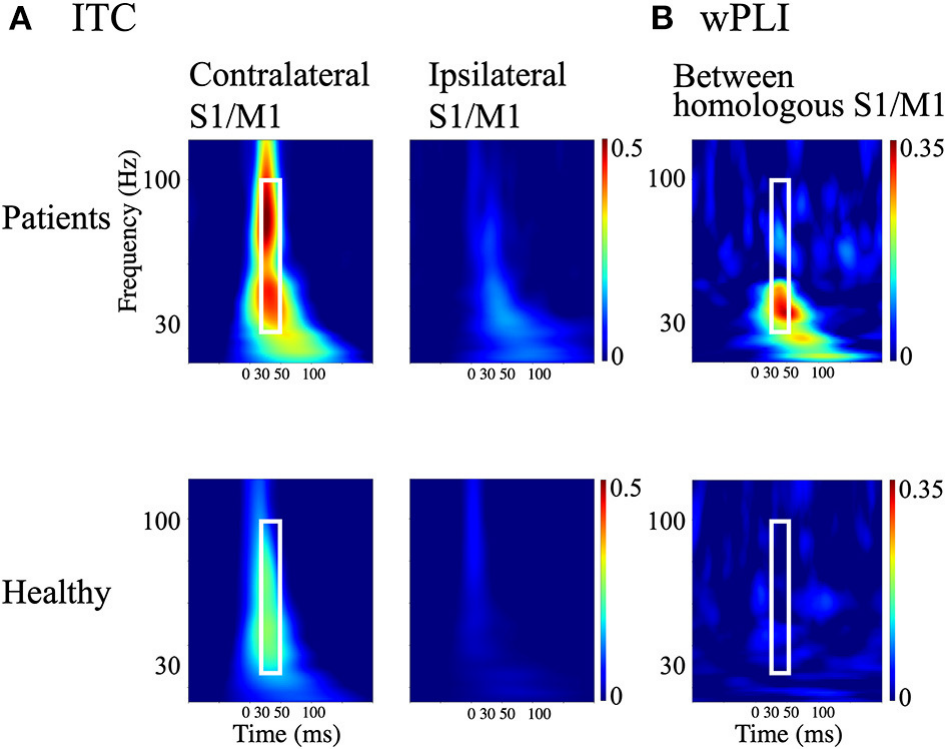
Measures of cortical synchrony following median nerve stimulation. **(A)** Time-frequency plots of grand-averaged intertrial phase coherence (ITC) in contralateral and ipsilateral S1/M1 regions of interest (ROIs; left and right panels, respectively). **(B)** Time-frequency plots of weighted phase-lag index (wPLI) between homologous S1/M1 ROIs. Upper panels: patients with bilateral C-reflex; lower panels: healthy controls. White rectangles show the time window of 30–50 ms and the frequency window of 30–100 Hz.

The time-frequency plots of the grand-averaged ITC showed prominent early (30–50 ms) intertrial phase synchrony in the frequency range of 30–100 Hz in the contralateral S1/M1 ROIs in both the patients and HCs (Figure 4A). The 30–50 ms time window identified using the ITC corresponded to the initial findings (see sections SEP and CR/BCR and Ipsilateral and contralateral SEF activity), where ipsilateral activity at around 36 ms was synchronized after P25m activity (at 27 ms) propagated with a CR-BCR time lag of 8 ms. Thus, we computed the average of the wPLI over the time-frequency window of 30–50 ms and 30–100 Hz for all subjects. The grand-averaged wPLI between the homologous S1/M1 ROIs was larger in the patients than in HCs within this time-frequency window (*p* = 0.004; Figure 4B). The wPLI for baseline (−100–0 ms) was not significantly different between the groups (*p* = 0.16).

**Figure 5:**
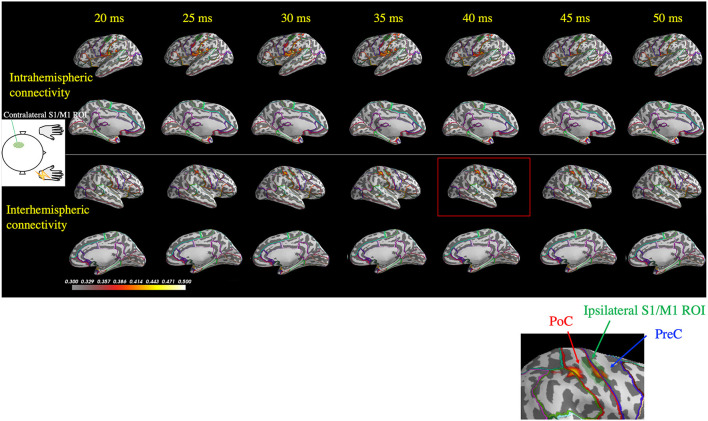
Spatiotemporal distribution of the weighted phase-lag index (wPLI) between the contralateral S1/M1 region of interest (ROI; green shaded area in the left hemisphere) and the rest of the cortex following right median nerve stimulation (Patient 2). The wPLI was averaged over the 30–100 Hz frequency window, which was identified using the intertrial phase coherence (see Figure 4A). Top two panels: interhemispheric connectivity; bottom two panels: intrahemispheric connectivity. Interhemispheric connectivity was distinct in the time range of 30–50 ms, especially in the S1/M1 ROI, whereas intrahemispheric connectivity showed a different pattern in the temporoparietal region. The inset shows a magnified view of the ipsilateral S1/M1 region at 40 ms, which corresponds to the red rectangle. PoC, postcentral gyrus; PreC, precentral gyrus.

Figure 5 depicts representative wPLI results of one patient's (Patient 2) response to right median nerve stimulation and shows the evaluation of the connectivity between the contralateral S1/M1 ROI (left hemisphere) and all cortical locations used in the MEG source estimation. Interhemispheric connectivity (Figure 5, lower two panels) was distinct, especially around the homologous S1/M1 ROI (highlighted in the inset figure) in the time range of 30–50 ms. Similar to the findings in the previous section, the distinct time courses of the spatial patterns of wPLI in the right and left hemispheres suggest that the interhemispheric connectivity results were not caused by artificial cross-talk in the MEG source estimates.

Analysis of the average wPLI for the time-frequency window of interest (30–50 ms and 30–100 Hz) between the contralateral S1/M1 ROI and all DK regions revealed significantly higher values in the patients than in HCs for the homologous ROI, precentral gyrus (PreC), postcentral gyrus (PoC), and other regions interhemispherically. Interestingly, the intrahemispheric IP connection was also revealed as highly significant. All statistically significant connections are listed in Supplementary Table 1.

**Figure 6:**
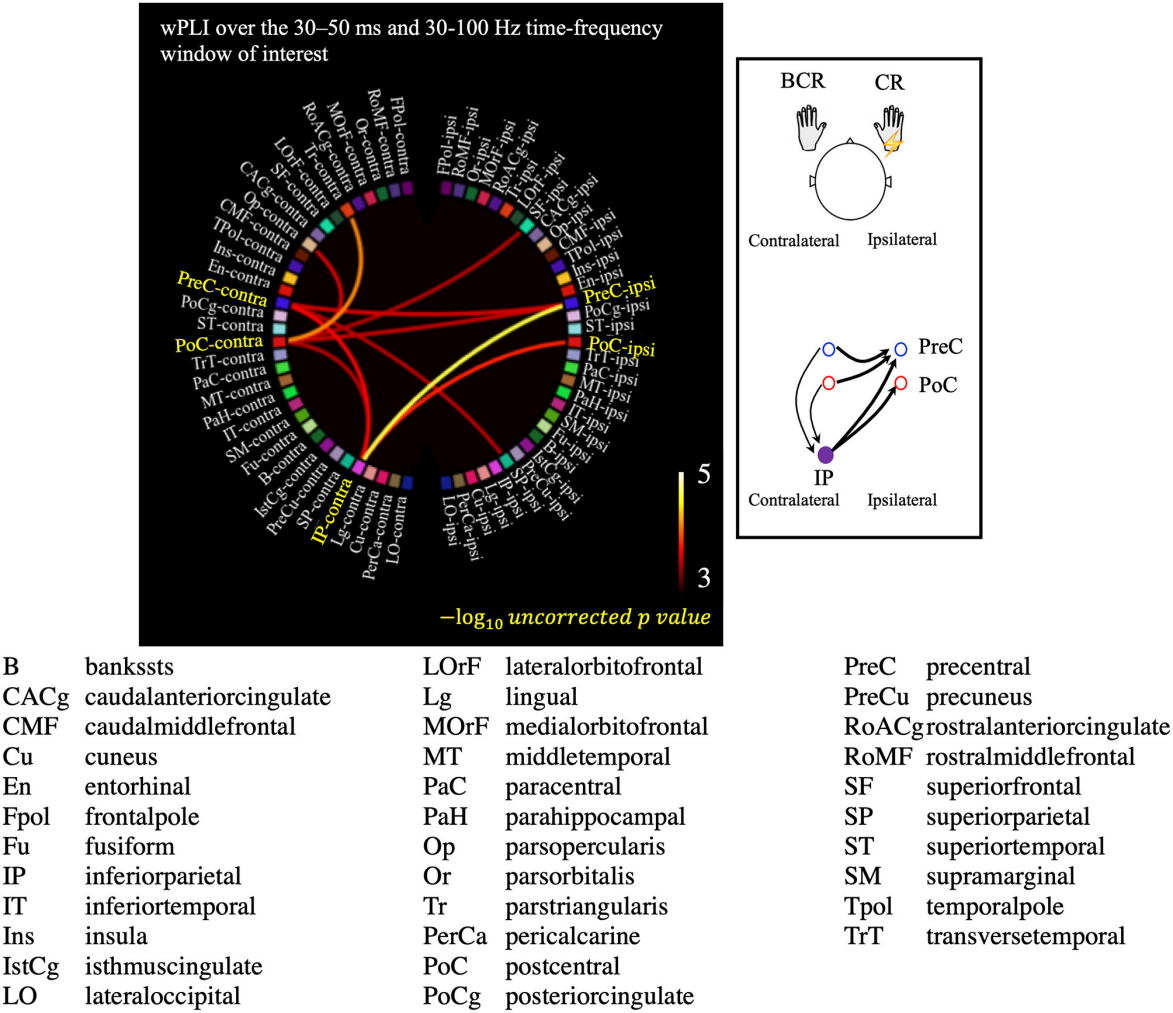
Results of the all-to-all connectivity analysis visualized on a circular representation, showing only the connections involving the S1/M1 regions. Connectivity was measured using the averaged weighted phase-lag index value over the 30–50 ms and 30–100 Hz time-frequency window of interest. A negative 10-based logarithm of uncorrected p values (uncorrected *p* < 5 × 10^−4^) is indicated by the color bar. The schematic image of significant connections that contains each of PreC, PoC, and IP is shown in the subfigure.

The all-to-all connectivity analysis revealed significant connectivity between several pairs of regions in which S1/M1 was included: PreC-PreC, PoC-PreC, IP-PreC, and IP-PoC interhemispherically (contralateral region-ipsilateral region), and PreC-IP and PoC-IP intrahemispherically (contralateral region-contralateral region; Figure 6). All statistically significant connections are listed in Supplementary Table 2. These results suggest that in the patients with BCR the contralateral S1/M1 was strongly connected to the ipsilateral M1 at 30–50 ms via the contralateral IP.

## Discussion

The presence of BCR provides concrete neurophysiological evidence that bilateral M1 are strongly involved in the response to unilateral somatosensory input. Our results revealed bilateral SEF activity (Figures 2B, 3) and enhanced interhemispheric connectivity (Figures 4–6) in patients with BCR. The time delay between contralateral and ipsilateral activity corresponded to the CR-BCR time lag (Table 2). The early enhanced connectivity between contralateral S1/M1 and ipsilateral M1 occurred within 50 ms, which was mediated by the contralateral IP (Supplementary Tables 1, 2). The MEG results provide novel insights into the pathophysiological mechanism underlying BCR, suggesting that homologous S1/M1 are strongly connected, probably transcallosally, and that the IP mediates the transcallosal connectivity.

### Cross-Talk Is Prominent in the Presence of a Giant SEP

Previous EEG studies have shown ipsilateral SEP activity in patients with BCR. Shibasaki et al. (3) observed a CR-BCR time delay of 9–11 ms in four out of eight patients with progressive myoclonic epilepsy, and Wilkins et al. (10) found a 10-ms delay in one out of seven Alzheimer's disease patients. Ikeda et al. (12) reported a 9-ms delay in one out of two patients with cortical tremor, and Brown et al. (11) comprehensively investigated BCR and reported in three out of nine patients with cortical myoclonus a delay of 10–16 ms. In these EEG studies, ipsilateral hemispheric activity was also observed with a time delay of 9–15 ms (3) and 9–18 ms (11), respectively, from the peak of the contralateral P25 to that of the ipsilateral homologous component. In the present study the cortical time delay was 7–12 ms. The wide variability in the cortical time delays could be due to differences in the patient populations among the studies and, perhaps more importantly, to differences between EEG and MEG in their sensitivity to specific source components of the evoked response. As shown in Figure 1, ipsilateral activity measured using EEG was less clear because of volume conduction effects related to the giant component (4) and limited spatial resolution (53, 54). EEG waveforms can be a mixture of overlapping scalp potentials generated by bilateral activity (55). Thus, investigations of ipsilateral activity as well as whole-brain connectivity using EEG is challenging with the presence of prominent contralateral activity (i.e., a giant component of SEP). To the best of our knowledge, no previous studies have investigated bilateral SEF activity with a giant component in myoclonus patients. In the current study using MEG, the spatiotemporal distribution of the estimated ipsilateral activity was clearly spatially distinct from that of contralateral activity (Figures 2B, 3). This suggests that the MNE source localization can ameliorate leakage effects (56, 57), thereby making it possible to dissociate ipsilateral activity from the giant contralateral activity.

The difference in the observed cortical time delay (9.4 ms) and the CR-BCR onset time lag (8.4 ms) may be due to the use of the peak latencies of the cortical responses. Measuring the onset rather than the peak times of the motor cortex activity in each hemisphere might provide a delay time closer to that observed for the CR-BCR time lag. However, because contralateral N20m and P25m are close in time and space, it is very difficult to determine the precise onset of the P25m reliably.

As an alternative approach to modeling the bilateral S1/M1 sources, we also attempted to use a double ECD model (58). However, we found a good fit in only one patient; presumably the small magnitude of the ipsilateral S1/M1 sources made the two-dipole fitting unstable in our cases. An advantage of distributed source models like the MNE is that only minimal assumptions are required; for example, there is no need to specify a priori the number of sources. For the localization of contralateral S1, MNE of SEP has been found to provide accuracy comparable to that obtained with ECD (59).

### Ipsilateral Activity in Patients With BCR as a Homologous Motor Response of the Contralateral Giant Component

The precise generator source of the giant SEP/SEF has not been fully elucidated; however, motor cortical hyperexcitability has been suggested to be involved (6, 7, 60, 61). Specifically, in a transcranial magnetic stimulation (TMS) study using short-interval intracortical inhibition, Hanajima et al. (61) suggested a pathological mechanism in patients with cortical reflex myoclonus whereby inhibitory GABAergic interneurons of the motor cortex are directly affected.

Our findings suggest that ipsilateral activity is homologous to the giant P25m component. First, the time difference between the peak latency of the giant P25m and CR onset was 10.7 ± 2.1 ms. This time difference indicates the conduction time from the contralateral M1 to the stimulated muscle, in response to the electrical stimulation (1, 3, 5, 9, 13, 62). Similarly, the time difference between the peak latency of ipsilateral activity and BCR onset was 9.7 ± 3.3 ms. This time difference is assumed to correspond to the conduction time from the ipsilateral M1 to the non-stimulated muscle associated with BCR.

Second, the connectivity analysis indicated a strong connection between the homologous motor cortices in patients with BCR (Figure 6; Supplementary Tables 1, 2). Significant connections were revealed interhemispherically between PreC–PreC and PoC–PreC, but not between PoC–PoC (contralateral region-ipsilateral region, Figure 6 and Supplementary Table 2). This finding is compatible with a previous report by Terada et al. (63), which suggested that there is no interhemispheric connection between bilateral somatosensory areas in humans. Instead, bilateral motor cortices may be strongly related to BCR. Sensory inputs to the M1 have been suggested to be closely associated with the performance of the opposite M1 transcallosally (13, 64, 65). It is generally accepted that the transcallosal connection is inhibitory (66); interhemispheric inhibition of TMS is mediated by a facilitatory transcallosal population synapsing onto a local inhibitory population in the motor cortices (67), and the local deficit in inhibitory GABAergic neurons was shown in the motor cortices of patients with cortical reflex myoclonus (61). On the basis of inhibitory transcallosal connections, we hypothesize that the enhanced connection between the bilateral motor cortices may be compensating for the physiological inhibitory connection of hyperexcitable motor cortices in patients with BCR. This could be confirmed in a future TMS study.

### Ipsilateral Activity Within 50 ms in Healthy Controls Cannot Be Detected Reliably

HCs did not show prominent ipsilateral SEF activity within 50 ms (Figure 2B). Bilateral receptive fields have been reported in non-human primates (68). In the human brain, various approaches have been used to search for an equivalent bilateral representation of somatosensory information at the lower level. These approaches included SEP/SEF (39–41, 69–75) and fMRI (76, 77). However, these studies demonstrated that detection of ipsilateral responses in humans is highly variable and are not reliably found in the left or right hemispheres (42). Early physiological ipsilateral SEP/SEF activity is weak and is difficult to detect reliably using sensor-space analysis, which is susceptive to volume-conduction (41, 69, 70, 73–75), or source-based analysis, which relies on a complete source model (39, 40, 71, 72). Moreover, most results showed ipsilateral activity after 50 ms. Considering that S2 activation arises after 50 ms, it remains controversial whether somatosensory information carried by the median nerve reaches lower level sensorimotor areas of both the ipsilateral and contralateral hemispheres within 50 ms. In one paper that used blind source decomposition (42), ipsilateral SEP activity within 50 ms was observed in healthy subjects in both the left and right hemispheres. Therefore, we believe that our results of ipsilateral activity in patients with BCR represent excessive enhancement of the physiological components of normal ipsilateral activity, rather than occurrence of an abnormal component. This assumption is consistent with previous studies of giant SEPs, which suggest that a giant contralateral SEP may result from pathological enhancement of certain cortical components of a normal SEP (4, 78).

### Pathophysiology of Initiation of BCR via a Hyperexcitable Transcallosal Pathway

Possible pathways initiating the BCR include the transcallosal pathway, direct input to the ipsilateral M1, the thalamic ascending projection, and top-down inputs from S2. Our findings suggest that the transcallosal pathway is the most likely (3, 10, 11). First, direct peripheral input to ipsilateral M1 and direct input from the contralateral nucleus of thalamus are unlikely because these pathways cannot explain the CR-BCR time lag or cortical delay between the ipsilateral and contralateral hemispheres. Kanno et al. (39) reported two epilepsy patients who showed ipsilateral SEF activity without CR/BCR. These patients who had severe left hemispheric damage showed no contralateral activity in response to right median nerve stimulation, however, they showed ipsilateral activity in S1. The authors suggested that the ipsilateral activity was due to direct peripheral input to the ipsilateral S1. However, ipsilateral activity in their study occurred after 50 ms. Thus, this abnormal ipsilateral response differs from the activity related to BCR. Second, given that S2 displays initial activation at around 60–70 ms after stimulation (43), the early spread of cortical excitation in BCR occurring within 50 ms is too early to be consistent with top-down inputs from S2 (43). Furthermore, in patients with cortical reflex myoclonus, excitability of S2 is not pathologically enhanced (6). Thus, the involvement of S2 is unlikely to be the pathway of BCR. Instead, the transcallosal pathway is the most reasonable explanation for the initiation of BCR. Moreover, this is compatible with the aforementioned physiology of inhibitory transcallosal connection.

### The Modulating Role of IP in Disinhibition of Transcallosal Inhibitory Process

Our results suggest that the bilateral representation of sensorimotor responses is associated with pathologically enhanced disinhibition of transcallosal inhibitory processes within M1 cortices. In addition to the connections between homologous S1/M1, connectivity (i.e., wPLI) was significantly enhanced between the contralateral IP and bilateral S1/M1 (Figure 6; Supplementary Tables 1, 2). These results suggest that the contralateral IP mediates BCR by involving bilateral S1/M1. The healthy motor cortex orchestrates movement, and it is likely that transcallosal inhibition acts to transform elemental mass movement into a meaningful pattern of synergistic activity. Upon receiving a movement command from higher centers, i.e., IP, this cortical inhibition enables an appropriate output to be produced and inappropriate movements to be suppressed (79). IP is crucial for sensorimotor transformation (80–82) and contains a rich variety of transcallosal neurons that are responsive to different sensory stimuli that discharge in association with different types of movements (83, 84). Moreover, it has a physiological facilitatory transcallosal connection to bilateral M1 (67, 85). Therefore, IP may have an important role on controlling motor movements seen in BAFME patients with BCR. The wPLI is a correlation-based measure that as such cannot determine whether the involvement of IP is direct or indirect. However, it is reasonable to assume that the involvement of IP is indirect: the primary contribution in BCR is likely to be the interhemispheric connection between bilateral S1/M1. IP may have a secondary or modulating role in BCR. Based on this assumption, we propose that modulation of IP excitability may be beneficial for controlling BAFME symptoms. This should be investigated in a prospective study using non-invasive TMS.

### Limitations

The stimulus parameters were not consistent among subjects or within subjects (SEP and SEF) because SEP and SEF were measured with different clinical purposes (long latency vs. short latency). AEDs may have affected SEP/SEF and connectivity analyses. Several studies have reported no significant differences in SEP during treatment with AEDs (86–88); however, one study showed suppressed amplitude of giant SEPs under AEDs (12). Patient 2 was treated with a sodium channel blocker sometimes worsening the myoclonus. Since replacement to other AEDs had worsened the myoclonus, we continued the current regimen. Therefore, drug naïve patients would be desirable.

The rarity of the BCR caused several concerns. First, the number of participants were small. We disregarded hemispheric dominance and concatenated the conditions for analysis to obtain significant statistics (7 vs. 30 arms). The wPLI results may have been affected by hemisphere dominance when the IP, a higher cognitive area, was involved. However, we found minimal differences in wPLI from the IP between the dominant and non-dominant hemispheres. Second, the types of participants in the current retrospective study were limited to BAFME patients among the cortical reflex myoclonus. A recent study suggested that the cortical excitability of BAFME may be different from that of other non-BAFME diseases (89). Thus, the current findings may be more specific to BAFME rather than other diseases with cortical reflex myoclonus in general. Therefore, more patients need to be recruited to fully investigate the general pathophysiology of BCR.

### Conclusions

The current MEG results confirmed bilateral SEF activity in patients with BCR and suggested that the transcallosal pathway is the probable pathway that initiates BCR. Disinhibition of transcallosal inhibitory processes within M1 cortices were related to the bilateral representation of sensorimotor responses. Hyperexcitable motor cortices were mediated by the contralateral IP.

## Data Availability Statement

The raw data supporting the conclusions of this article will be made available by the authors, without undue reservation.

## Ethics Statement

The studies involving human participants were reviewed and approved by Ethical Committee of Kyushu University Hospital. The patients/participants provided their written informed consent to participate in this study.

## Author Contributions

TMa and ST: study conception and design. TMa, KH, and HS: data collection. TMa, SA, and TMi: analysis and interpretation of results. TMa: draft and manuscript preparation. YG and SS: revision of manuscript. All authors approved the final version of the manuscript.

## Funding

This work was supported by JSPS KAKENHI Grant No. JP20J00552, Nakatani Foundation for advancement of measuring technologies in biomedical engineering, The Japan Epilepsy Research Foundation, The Osaka Medical Research Foundation for Intractable Diseases and the National Institute on Deafness and other Communication (Grant No. R01DC016765) and NIH (Grant Nos. R21NS101373, R01NS104116, R01NS069696, and S10ODRR031599).

## Conflict of Interest

The authors declare that the research was conducted in the absence of any commercial or financial relationships that could be construed as a potential conflict of interest.

## Publisher's Note

All claims expressed in this article are solely those of the authors and do not necessarily represent those of their affiliated organizations, or those of the publisher, the editors and the reviewers. Any product that may be evaluated in this article, or claim that may be made by its manufacturer, is not guaranteed or endorsed by the publisher.

## Abbreviations

AED: antiepileptic drug
BAFME: benign adult familial myoclonus epilepsy
BCR: bilateral C-reflex
CR: C-reflex
DK Atlas: Desikan-Killiany Atlas
dSPM: dynamic statistical parametrical mapping
ECD: equivalent current dipole
HC: healthy control
IP: inferior parietal cortex
ISI: interstimulus interval
ITC: intertrial phase coherence
JLA: jerk-locked back averaging
MEG: magnetoencephalography
PoC: postcentral gyrus
PreC: precentral gyrus
ROI: region of interest
SD: standard deviation
SEF: somatosensory-evoked field
SEP: somatosensory-evoked potential
S1/M1: primary sensorimotor cortex
S2: secondary somatosensory cortex
wPLI: weighted phase-lag index.
