# Supplementary material for: Bilateral Representation of Sensorimotor Responses in Benign Adult Familial Myoclonus Epilepsy: An MEG Study
Source: Front Neurol. 2021 Oct 26;12:759866. doi: 10.3389/fneur.2021.759866 (PMC8577121; doi:10.3389/fneur.2021.759866)
Supplement: Supplementary file 1 [file Data_Sheet_1.docx]

**Supplementary Table 1. Connectivity analysis (functional ROIs)**

| Contra S1/M1 ROI vs. all DK regions plus ipsi S1/M1 ROI (corrected *p* value) | |
| --- | --- |
| Interhemispheric  (contra S1/M1 ROI-ipsilateral region) | **Homologous** (0.004) |
|  | B (0.01) |
|  | CMF (0.009) |
|  | En (0.008) |
|  | Fu (0.004) |
|  | IT (0.005) |
|  | IstCg (0.008) |
|  | LO (0.04) |
|  | Lg (0.03) |
|  | PaH (0.006) |
|  | **PoC** (0.004) |
|  | PoCg (0.01) |
|  | PreCu (0.02) |
|  | **PreC** (0.004) |
|  | SF (0.009) |
|  | SP (0.009) |
|  | ST (0.01) |
|  | TPol (0.03) |
|  | TrT (0.01) |
| Intrahemispheric  (contra S1/M1 ROI-contralateral region) | B (0.009) |
|  | En (0.03) |
|  | Fu (0.009) |
|  | **IP** (0.007) |
|  | IT (0.03) |
|  | LO (0.009) |
|  | LOrF (0.01) |
|  | MT (0.009) |
|  | PaC (0.03) |
|  | PaH (0.01) |
|  | Op (0.009) |
|  | Tr (0.01) |
|  | PoC (0.01) |
|  | PreC (0.04) |
|  | RoACg (0.01) |
|  | SF (0.03) |
|  | ST (0.02) |
|  | SM (0.02) |
|  | SP (0.03) |
|  | FPol (0.03) |
|  | TrT (0.009) |

S1/M1, primary sensorimotor cortex; ROI, region of interest; DK, Desikan-Killiany; B, bankssts; CMF, caudalmiddlefrontal; En, entorhinal; Fu, fusiform; IP, inferiorparietal; IT, inferiortemporal; IstCg, isthmuscingulate; LO, lateraloccipital; LOrf, lateralorbitofrontal; Lg, lingual; MT, middletemporal; PaH, parahippocampal; PoC, postcentral; PoCg, posteriorcingulate; PreCu, precuneus; PreC, precentral; SF, superiorfrontal; SP, superiorparietal; ST, superiortemporal; Tpol, temporalpole; TrT, transversetemporal. Bold texts are highlighted in the manuscript.

**Supplementary Table 2. Connectivity analysis (all-to-all)**

| All-to-all (corrected *p* value) | |
| --- | --- |
| Interhemispheric  (contralateral region-ipsilateral region) | **IP-PoC** (0.05) |
|  | **IP-PreC** (0.04) |
|  | IP-SF (0.04) |
|  | Lg-SF (0.05) |
|  | **PoC-PreC** (0.05) |
|  | PoC-SF (0.05) |
|  | **PreC-PreC** (0.05) |
|  | PreC-SP (0.05) |
|  | SF-PoC (0.05) |
| Intrahemispheric  (contralateral region-contralateral region) | B-CMF (0.05) |
|  | IP-MT (0.05) |
|  | LO-PaC (0.05) |
|  | LO-PerCa (0.05) |
|  | **PoC-IP** (0.05) |
|  | Op-PoC (0.05) |
|  | Tr-PoC (0.05) |
|  | **PreC-IP** (0.05) |
|  | PreC-RoACg (0.05) |
|  | MT-TrT (0.05) |

B, bankssts; CMF, caudalmiddlefrontal; IP, inferiorparietal; LO, lateraloccipital; Lg, lingual; MT, middletemporal; PaC, paracentral; Op, parsopercularis; Tr, parstriangularis; PerCa, pericalcarine; PoC, postcentral; PreC, precentral; RoACg, rostralanteriorocingulate; SF, superiorfrontal; SP, superiorparietal; TrT, transversetemporal. Bold texts are highlighted in the manuscript.
